# Evolving computational paradigms for noncoding variant pathogenicity prediction

**DOI:** 10.3389/fmolb.2026.1761673

**Published:** 2026-04-30

**Authors:** Beibei Wang, Siyuan Song, Song Cheng, Yihang Lin, Liang Yu, Yan Li, Xiang Chen

**Affiliations:** 1 School of Management, Xi’an Polytechnic University, Xian, Shaanxi, China; 2 Yangtze Delta Region Institute (Quzhou), University of Electronic Science and Technology of China, Quzhou, Zhejiang, China; 3 School of Computer Science and Technology, Xidian University, Xian, Shaanxi, China; 4 School of Computer Science and Technology, Xinjiang University, Urumqi, Xinjiang, China; 5 School of Opto-electronic and Communication Engineering, Xiamen University of Technology, Xiamen, Fujian, China

**Keywords:** clinical translation, computational modeling, genome language models, noncoding variants, pathogenicity prediction

## Abstract

The rapid expansion of whole-genome sequencing (WGS) has highlighted the important contribution of noncoding variants to human disease, yet their pathogenic mechanisms remain difficult to resolve. Traditional statistical and experimental approaches often struggle to capture complex regulatory interactions or establish causal links, leaving many noncoding variants classified as variants of uncertain significance in clinical databases. Recent advances in computational modeling have substantially improved pathogenicity prediction by integrating genomic, epigenetic, and structural information. In parallel, genome language model (gLM)-inspired methods have enabled more context-aware interpretation of noncoding sequences and improved model generalization. This review summarizes current computational approaches, data modalities, and evaluation strategies for noncoding variant pathogenicity prediction, discusses key challenges in interpretability and data heterogeneity, and highlights emerging opportunities for clinical translation.

## Introduction

The vast noncoding regions of the human genome remain largely unexplored as genetic studies have historically focused on protein-coding variants (Alexander et al., 2010). Emerging evidence shows that most disease-associated variants are located in noncoding regions (Wu et al., 2024); these variants usually exert their effects by disrupting transcriptional regulation, RNA processing, and chromatin organization (Zhang and Lupski, 2015), thereby contributing to the onset and progression of complex disorders such as cancer (Fredriksson et al., 2014; Khurana et al., 2016), cardiovascular disease, and neurodegenerative disease (Spielmann and Mundlos, 2016; Nemeth et al., 2024). Accordingly, understanding how noncoding variants affect disease is essential for improving our knowledge of disease mechanisms and for advancing precision medicine (Ellingford et al., 2022; Ruffo et al., 2025).

Genome-wide association studies (GWASs) (Visscher et al., 2012; Jin et al., 2022; Schipper and Posthuma, 2022) identify statistical associations between genetic variants and phenotypic traits across large populations. GWASs have been used to successfully discover genetic loci associated with disease; however, their direct clinical interpretation is limited. Because of linkage disequilibrium (LD), where a signal often represents a block of correlated variants rather than a single causal site, fine mapping is required (Uffelmann et al., 2021). To address this issue, there has been an increase in research studies embedding GWAS signals with functional genomic annotation, as well as applying probabilistic fine-mapping methods to give greater priority to noncoding variants with putative regulatory function (Li and Zhou, 2025). Meanwhile, the widespread adoption of whole-genome sequencing (WGS) in both research and clinical practice has further expanded the scale of the interpretation problem (Bagger et al., 2024; Brlek et al., 2024; Qiao et al., 2024); each genome contains millions of mostly noncoding variants, making systematic interpretation challenging (Austin-Tse et al., 2022). Both GWAS fine mapping and clinical diagnostics increasingly rely on computational frameworks that can rank and score noncoding variants to prioritize mechanistically informative and clinically relevant candidates. Noncoding variant interpretations are observed between clinical diagnoses and GWAS fine mapping. In clinical settings, the main goal is to identify pathogenic variants, particularly those with effects large enough to be demonstrable with a clear link from the phenotype to the tissue/development period of interest. These types of variants are typically assessed in the context of family segregation, functional studies, and curated clinical databases (Ellingford et al., 2022). By contrast, GWAS is mainly concerned with identifying loci that influence molecular or organismal traits, where the underlying variants often have modest effects and do not necessarily correspond directly to pathogenic alleles. Consequently, predictive models developed for clinical pathogenicity assessment and those designed for GWAS fine mapping should not be treated as interchangeable because they are built to address different biological questions and must be evaluated using different criteria.

Functional and pathogenic effects are closely related and are often conflated. Both are typically measured using individual variant assays, but their biological meanings differ. Functional effects refer to the direct perturbation of molecular phenotypes, such as altered transcription factor (TF) binding, changes in gene expression, or splicing disruption, and therefore represent proximal mechanistic signals (Wang X. et al., 2024). By contrast, pathogenic effects depend on whether these molecular alterations occur in disease-relevant tissues or developmental contexts and translate into disease phenotypes or risks (Guo et al., 2024; Huang et al., 2024; Huang et al., 2025). Importantly, the relationship between the two is not strictly dichotomous. Many sequence-based models predict regulatory-layer molecular effects rather than pathogenicity, yet their outputs still provide informative clues about disease relevance. In particular, recent unified frameworks such as AlphaGenome (Avsec et al., 2026), which jointly model functional consequences across multiple regulatory layers, have further strengthened the ability of functional predictions to indicate disease relevance, thereby forming an important bridge between functional effect prediction and direct pathogenicity inference. At the same time, it should be emphasized that many variants can produce measurable functional effects without necessarily causing disease. This is particularly relevant for complex traits, where clinical significance arises from the cumulative effects of multiple small-effect variants (Lappalainen et al., 2024; Xu et al., 2025).

This conceptual distinction helps explain why early studies relied on indirect markers and chains of evidence to identify candidate noncoding variants (Ritchie et al., 2014); this reliance was driven by two major constraints: the scarcity of confidently validated pathogenic labels and the strong context dependence of noncoding regulatory mechanisms. In practice, candidate variants were first filtered according to the population frequency, evolutionary conservation, and heuristic rules based on functional genomic regions (Ward and Kellis, 2012) and were then further annotated using resources such as ENCODE (ENCODE Project Consortium, 2012) and Roadmap Epigenomics (Fisher et al., 2015) to obtain mechanistic clues. Pathogenicity was subsequently inferred by integrating these annotations with functional validation results and other lines of supporting evidence. However, the evidence-based method was limited in important ways, including wide variability between signals with respect to scale, resolution, and biological relevance; many annotations were made at the region level rather than at the single-variant level; and accurate mapping to disease-relevant tissues or cell types was often lacking, leading to potential false positives and false negatives. Even as large-scale projects expanded functional annotation catalogs, relevant signals could still be missed.

Against this background, the rapid development of artificial intelligence (AI), machine learning (ML), and especially deep learning (DL) provided a new framework for noncoding variant prediction. By automatically extracting informative features and modeling complex nonlinear relationships, these methods substantially improved the ability to prioritize potentially deleterious variants. More recently, general language model (gLM)-inspired approaches (Shu et al., 2026) that treat genomic sequences as biological language have further expanded model representational capacity and improved predictive performance in some contexts. Current methods that are used to evaluate the effect of noncoding variants can generally be categorized into two classes of computational approaches. The first strategy infers disease relevance indirectly from predicted functional effects (Wang X. et al., 2024); models predict impacts on molecular phenotypes such as splicing, gene expression, chromatin states, or TF binding and use these predictions to prioritize potentially pathogenic variants, with the direct output being functional consequences rather than pathogenicity labels. Enformer (Avsec et al., 2021) and the more recent AlphaGenome (Avsec et al., 2026) both fall into this category. The second strategy predicts pathogenicity more directly under pathogenicity-oriented supervision. Its training endpoints are typically derived from manually curated resources such as ClinVar (Landrum et al., 2025) or from constructed pathogenic/benign label sets. These models learn a mapping from multisource input features to an integrated pathogenicity score, making them more closely aligned with the practical needs of clinical interpretation. Combined Annotation-Dependent Depletion (CADD) (Schubach et al., 2024) is a representative example of this strategy. In the following sections, we primarily focus on the development of the second category of methods.

## Noncoding variants as drivers of human disease

### Mapping the functional hierarchy of noncoding regions in the genome

**FIGURE 1:**
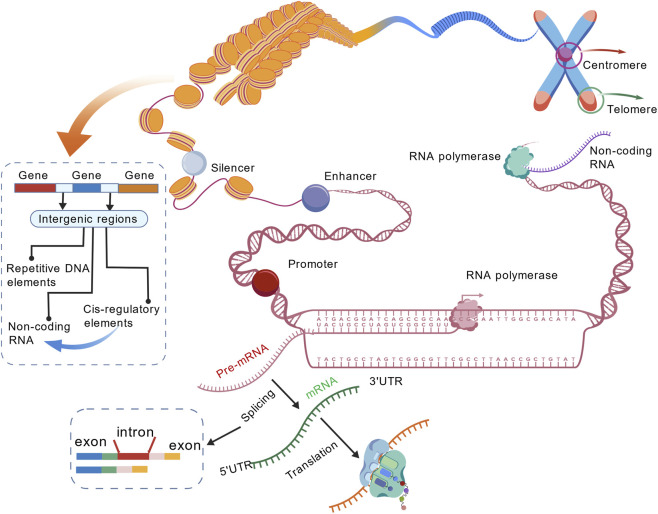
Schematic representation of noncoding regions in the human genome, from chromosomes to DNA sequences, including key functional elements such as promoters, enhancers, silencers, 5′UTRs, 3′UTRs, introns, telomeres, centromeres, and repetitive DNA elements. During transcription, introns are spliced out to generate mature mRNA. Noncoding RNAs are transcribed throughout the genome by RNA polymerase and primarily include long noncoding RNAs (lincRNAs), microRNAs (miRNAs), and circular RNAs (circRNAs). Telomeres and centromeres are annotated on the chromosomes. Intergenic regions, located between two genes, such as between exons and introns, contain regulatory elements (such as enhancers and silencers), repetitive DNA elements (such as transposons and pseudogenes), and noncoding RNA genes. Noncoding RNAs are transcribed under the action of these *cis*-regulatory elements.

Noncoding regions refer to segments of the genome that are not translated into proteins. Although once termed “junk DNA,” they are now widely recognized as having diverse and important biological functions (Walter, 2024). With the advancement of whole-genome sequencing (WGS) technologies, increasing evidence has shown that most disease-associated genetic variants reside in noncoding regions, where they play critical roles in transcriptional regulation, RNA processing, and chromatin architecture. Based on their location and function, noncoding regions can be classified into the following categories (Figure 1).

#### *Cis*-regulatory elements

These include promoters, enhancers, silencers, and insulators (Encode Project Consortium, 2012). Promoters can be found upstream from the starting position of transcription and are used mainly for binding of RNA polymerase and TFs (Whitfield et al., 2012; Haberle and Stark, 2018). Enhancers and silencers can also exist at greater distances from the target gene, enhancing or repressing transcription of target genes via protein interactions (Panigrahi and O’Malley, 2021; Pang et al., 2023). Insulators form chromatin boundaries, blocking enhancers from acting on non-target promoters and preventing heterochromatin spread (Brasset and Vaury, 2005). In addition, intergenic regions also contain these *cis*-regulatory elements, which play a crucial role in the transcription of noncoding RNAs.

#### Post-transcriptional regulatory regions

These mainly include the 5′ untranslated region (5′UTR) and the 3′ untranslated region (3′UTR) of messenger RNA (mRNA) (Chu et al., 2024). They play vital roles in regulating translation efficiency, mRNA stability, and microRNA (miRNA) binding (Bohn et al., 2023). Models such as CADD (Schubach et al., 2024) and FINSURF (Moyon et al., 2022) predict the pathogenicity of variants found in the 5′UTR and 3′UTR by integrating measures of sequence conservation, epigenetic information, and functional annotation into assessments that can help determine their effect on gene expression and disease relevance.

#### Introns and splicing regulatory elements

Introns are the sequences separating exons that are removed during splicing. Deep intronic mutations can cause splicing to occur incorrectly, thereby affecting gene expression (Vaz-Drago et al., 2017; Barbosa et al., 2023). Some introns have regulatory functions because they contain transcription promoters, enhancers, or TF-binding sites (TFBSs). They contribute to transcriptional regulation by determining whether certain isoforms of mRNA are expressed using splicing activity. The DL model [i.e., DYNA (Zhan et al., 2025)], which uses a Siamese network, compares the splicing activities of wild-type versus variant sequences to accurately predict pathogenic intronic variants from splicing. TRAP (Gelfman et al., 2017), on the other hand, uses transcript information and sequence-based features, such as GERP++ (Davydov et al., 2010), to predict the impact of intronic variants on transcripts through ML methods, helping identify disease-associated intronic variants.

#### Noncoding RNAs

Noncoding RNAs (ncRNAs) can be considered to span the entire genome as they are widely distributed across different regions of the genome and play crucial roles in gene expression regulation, cellular functions, and genome stability (Tóth and Hannon, 2011). These include miRNAs, long noncoding RNAs (lncRNAs), circular RNAs (circRNAs), and transfer RNA (tRNA)-derived fragments. The miRNAs bind complementarily to mRNAs to regulate degradation or translation; lncRNAs (>200 nucleotides) participate in chromatin remodeling, transcriptional regulation, and RNA processing (Statello et al., 2021); circRNAs form covalently closed loops that resist RNase degradation and act as miRNA “sponges” or protein-binding platforms; tRNA-derived fragments may play roles in RNA interference or translation inhibition. The JARVIS model, which integrates genome-wide residual variation intolerance scores (gwRVIS) and genomic sequences, can effectively evaluate the impact of such variants on ncRNA function.

#### Structural and repetitive elements

These include tandem repeats, pseudogenes, transposons, telomeres, and centromeres. These regions are closely associated with genome stability, replication, and chromosomal architecture and represent important sources of genetic variation. DYNA not only focuses on splicing-related variants but is also capable of modeling variants that alter the 3D genome architecture or chromatin structure, such as enhancer rearrangements and structural variants in intergenic regions, effectively predicting whether such variants are disease-associated.

### Deciphering the functional impact induced by noncoding variants

Functional models for noncoding variants generally do not directly output clinical pathogenicity conclusions. Instead, they primarily predict the impact of variants on molecular phenotypes and are therefore better suited as tools for mechanistic interpretation and as sources of functional evidence for candidate variant prioritization. According to their central focus and the breadth of functional outputs they cover, functional models for noncoding variants can be broadly divided into three levels.

The first consists of specialized models centered on a single dominant modality, with each model focusing on the consequences of variation at one particular functional layer. Such models can capture only a subset of features and may overlook others. Representative splice-centric models include SpliceAI (Jaganathan et al., 2019), MMSplice (Cheng et al., 2019), and Pangolin (Zeng and Li, 2022). SpliceAI directly analyzes raw pre-mRNA sequences to predict splice sites and identify cryptic splice-altering variants; variants with high scores can often be validated at relatively high rates in RNA-seq data. MMSplice adopts a modular neural network architecture, separately modeling the effects of donor sites, acceptor sites, exonic sequences, and intronic sequences on splicing. It mainly learns quantitative changes in exon skipping and splicing efficiency and then combines these modules into an overall splicing impact score. Pangolin further emphasizes multi-tissue splicing prediction and incorporates cross-species training to better capture tissue-specific effects and variant impacts; its outputs represent predicted functional effects on splice-site usage. Beyond splicing, PromoterAI (Jaganathan et al., 2025) focuses on promoter variants that cause expression abnormalities. Using approximately 20 kb of the promoter-centered sequence context as input, it predicts the effect of promoter variants on gene expression. The model is first pretrained on multi-omic readouts and then fine-tuned using rare promoter variants from the Genotype-Tissue Expression (GTEx) project that are associated with linking promoter variation to abnormal expression. Basset (Kelley et al., 2016) primarily targets chromatin accessibility, using DNase-seq accessibility as the supervisory signal to train a convolutional neural network across 164 cell types; variant effects are then estimated through allelic differences, making it, in essence, a model that infers variant impact through the “accessibility channel.” ExPecto (Zhou et al., 2018) is mainly designed to predict tissue-specific expression changes. It first predicts a large number of epigenetic features from sequence and then uses spatial feature transformation together with a linear model to infer tissue-specific gene expression, thereby estimating variant-induced expression changes.

The second level includes models that allow a single framework to handle multiple modalities rather than relying on modality-specific specialized models. Multimodal models such as DeepSEA (Zhou and Troyanskaya, 2015), Basenji (Kelley et al., 2018), Enformer (Avsec et al., 2021), Sei (Chen et al., 2022), and Borzoi (Linder et al., 2025) have all demonstrated practical utility and broad applicability. One of the earliest representatives is DeepSEA, which directly predicts, from the sequence alone, large-scale chromatin features including TF binding, DNase I hypersensitive sites, and histone modifications and estimates variant effects through allelic differences. This represented an early attempt to jointly model multiple regulatory phenotypes within a single model. Basenji subsequently extended the input to much longer sequence contexts and jointly predicted DNase-seq, ChIP-seq, and CAGE coverage profiles, thereby beginning to connect local regulatory signals to broader expression-related effects. Enformer further advanced this line of work by jointly predicting gene expression and multiple epigenetic marks under a longer-context, multitask-learning framework, while leveraging attention mechanisms to improve long-range information integration and thereby enhance the prediction of variant-induced expression effects. Sei expanded this paradigm to much larger-scale chromatin prediction, covering tens of thousands of chromatin profiles and summarizing them into interpretable “sequence activity classes” to quantify how variants increase or decrease different regulatory activities, although its mechanistic interpretation remains largely confined to the chromatin layer. More recently, Borzoi has moved further toward a unified sequence-to-function framework by directly predicting RNA-seq coverage from DNA sequence and enabling the extraction of variant effects across multiple layers, including transcription, splicing, and polyadenylation. The general representations learned by these models make them amenable to rapid fine-tuning for new tasks; however, their broader generality may come at the cost of reduced performance on some specific tasks, and they often lack the depth of analysis achievable with modality-specific specialized models.

The third level consists of unified multimodal frameworks spanning multiple major functional categories. AlphaGenome (Avsec et al., 2026) is a representative example. It integrates multimodal prediction, long-range context modeling, and base-pair-resolution inference within a single framework to predict a broad range of genomic tracks across multiple cell types. Using a 1-Mb DNA sequence as input, AlphaGenome jointly predicts diverse functional readouts in one unified architecture, including gene expression, transcription initiation, splice-site usage and splice junctions, chromatin accessibility, histone modifications, transcription factor binding, and chromatin contact maps. In doing so, it can simultaneously characterize the molecular consequences of variants across multiple regulatory layers. Although AlphaGenome approaches disease relevance assessment, it remains fundamentally a functional effect predictor rather than a pathogenicity classifier. Its outputs can support disease-related interpretation, serving as a bridge between functional effect prediction and pathogenicity inference.

In summary, functional effect models focus on molecular phenotypes and are primarily suited for mechanistic interpretation rather than direct disease risk prediction. Even unified models such as AlphaGenome and Borzoi require integration with additional evidence—such as disease-relevant tissues, developmental stages, candidate target genes, and effect magnitude—to inform pathogenicity inference.

### Decoding the regulatory links between noncoding variants and disease

Noncoding variants can contribute to disease development through diverse molecular pathways, with varying types and functions validated in multiple disorders (Table 1). From a regulatory architecture perspective, many noncoding variants first manifest as local *cis*-regulatory perturbations, directly affecting regulatory elements within their genomic neighborhood, such as promoters, enhancers, silencers, or splicing regulatory sequences. The effectiveness of this localized regulatory mechanism makes it easier to associate these variants with nearby target genes. For example, enhancer variants can alter TF binding sites or disrupt enhancer–promoter spatial interactions, thereby deviating from normal gene expression patterns. Mutations in the enhancer upstream of the *TERT* promoter have been linked to malignancies such as melanoma and glioblastoma (Heidenreich and Kumar, 2017), whereas other diseases such as campomelic dysplasia are linked to reduced silencing activity of a mutation in the SOX9 silencer. Additionally, deep intronic mutations present in the SMN2 gene lead to splicing defects in patients diagnosed with spinal muscular atrophy (SMA) (Csukasi et al., 2019). By contrast, *trans*-regulation primarily depends on diffusible regulatory factors, such as miRNAs, which are capable of modulating the expression of multiple target genes over a broader range (Signor and Nuzhdin, 2018). Therefore, when noncoding variants affect the expression, processing, or activity of these *trans*-acting regulators, their consequences are often not confined to the local genomic environment but may instead propagate through broader regulatory networks, giving rise to more distal and systemic downstream effects. For example, mutations in the seed region of *miR-96* are associated with familial progressive hearing loss (Avraham et al., 2022). Beyond affecting gene expression through *cis*- or *trans*-regulatory mechanisms, noncoding variants can impact disease development through other forms of locally noncoding mechanisms. For instance, CGG repeat unit expansions in the 5′UTR are associated with fragile X syndrome (Broniarek et al., 2024), and HOTAIR variants within the promoter region are associated with breast cancer susceptibility (Milevskiy et al., 2016). Epigenetic modification-related variants may alter DNA methylation or RNA modification levels, exemplified by *MLH1* promoter methylation control variants linked to Lynch syndrome (Ward et al., 2013) and noncoding variants affecting METTL3-mediated m6A regulation in acute myeloid leukemia (Wen et al., 2023). In addition, copy number variations (CNVs) and structural rearrangements in noncoding regions have clinical importance; for instance, CNVs at 17p11.2 are associated with Smith–Magenis and Potocki–Lupski syndromes (Juyal et al., 1996; Potocki et al., 2007), and enhancer rearrangements located upstream of the TAL1 gene have been associated with T-cell leukemia (Mansour et al., 2014). Collectively, these examples highlight how different classes of noncoding variants can affect gene expression, RNA processing, epigenetic regulation, and chromatin architecture, underscoring the critical role of the noncoding genome in the study of genetic and complex diseases. In addition to these molecular mechanisms, the pleiotropic nature of regulatory elements and their positions within gene regulatory networks can influence the strength of selective constraint and their disease relevance. Variants affecting multiple target genes or located at hub positions in regulatory networks tend to experience stronger purifying selection and are, therefore, more likely to be associated with disease.

**TABLE 1:** Linking noncoding variant classes to molecular mechanisms and disease phenotypes.

Variant class	Molecular mechanism	Disease example
Enhancer variants	Alter TF binding sites; disrupt enhancer–promoter spatial interactions	*TERT* upstream enhancer mutations → melanoma and glioblastoma
Silencer variants	Weaken transcriptional repression	*SOX9* silencer mutations → campomelic dysplasia
RNA processing variants	(i) 5′UTR CGG repeat expansion → abnormal translation initiation; (ii) deep intronic mutations → aberrant splicing	(i) Fragile X syndrome; (ii) *SMN2* intronic mutations → spinal muscular atrophy (SMA)
Noncoding RNA variants	Affect miRNA seed sequence or lncRNA promoter activity	(i) *miR-96* seed region mutation → familial progressive hearing loss; (ii) *HOTAIR* promoter variants → breast cancer
Epigenetic modification-related variants	Alter DNA methylation or RNA modifications (e.g., m6A)	(i) *MLH1* promoter variants → Lynch syndrome; (ii) noncoding *METTL3* variants → acute myeloid leukemia
Copy number variations (CNVs) and structural rearrangements	Change the dosage or regulatory architecture	(i) CNVs at 17p11.2 → Smith–Magenis and Potocki–Lupski syndromes; (ii) Enhancer rearrangements upstream of *TAL1* → T-cell leukemia

This table provides an overview of the main types of noncoding variants, their molecular mechanisms, and examples of human diseases. For each variant class, we describe how alterations in regulatory or structural elements, including promoters, enhancers, silencers, UTRs, introns, telomeres, centromeres, and repetitive sequences, can disrupt gene expression or genomic stability. The affected molecular mechanisms include transcriptional regulation, RNA processing, chromatin structure, and higher-order genomic organization. Associated examples of diseases illustrate the clinical significance associated with each type of noncoding variant and further demonstrate the broad and widespread role of noncoding genomic variation in human pathophysiology.

## Key attributes underlying the pathogenic potential of noncoding variants

### An explicit attribute landscape of pathogenic noncoding variants

**FIGURE 2:**
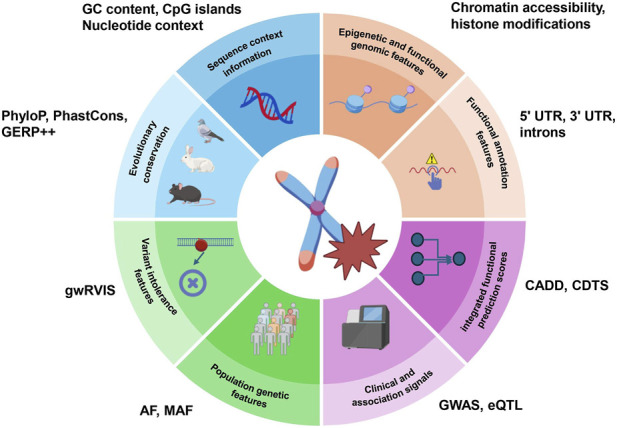
Potential attributes for predicting the pathogenicity of noncoding variants. This figure illustrates the overall framework for predicting the pathogenicity of noncoding variants. The central circle represents the pathogenicity of noncoding variants, while the inner circle displays the graphical representations of eight representative predictive attributes. The outer circle lists the specific characteristics or methods associated with these predictive attributes. The blank sections in the boxes represent the specific features of each attribute or method, further demonstrating how each attribute contributes to the assessment of noncoding variant pathogenicity. AF, allele frequency; AF is the frequency of a specific allele in the entire population. MAF, minor allele frequency; MAF is the frequency of the less common allele in the population.

The prediction of pathogenicity for noncoding variants relies on multidimensional biological attributes, which not only reveal the molecular functional changes that a variant may induce but also provide key input features for constructing prediction models (Figure 2). First, sequence context information (such as GC content, CpG islands, and nucleotide context) can reveal how the local sequence environment influences the sensitivity of variants. From an evolutionary perspective, signals relevant to noncoding variant pathogenicity should not be reduced to a single conservation score but rather viewed as multiple observable manifestations of selective constraint operating across different timescales. Cross-species conservation reflects long-term evolutionary constraint; highly conserved noncoding sequences often carry key regulatory functions, and variants occurring in these regions are, therefore, more likely to have biological consequences. Commonly used metrics include PhyloP (Pollard et al., 2010), PhastCons (Siepel et al., 2005), and GERP++ (Davydov et al., 2010). However, evolutionary information extends beyond conservation scores alone. Variant intolerance features, such as gwRVIS (Vitsios et al., 2021), can help identify depletion of variation and intolerance in functionally important regions along the human lineage and have been incorporated into models such as JARVIS (Vitsios et al., 2021) and NCBoost (Caron et al., 2019). Population genetic features, such as allele frequency (AF) derived from gnomAD and the 1000 Genomes Project (1kGP) (Auton et al., 2015), reflect recent or ongoing purifying selection: variants with stronger potentially deleterious effects are generally less likely to reach high frequency, resulting in an overall inverse relationship between allele frequency and effect size at the population level. Importantly, this relationship is statistical rather than absolute; rare variants are not necessarily pathogenic, and common variants are not necessarily functionally neutral. Therefore, conservation, allele-frequency spectra, and regional intolerance should be viewed as complementary forms of evolutionary evidence that together inform the prediction of noncoding variant pathogenicity. Next, epigenetic and functional genomic features, including experimentally derived genomic functional states and three-dimensional structural information, such as chromatin accessibility (by DNase-seq/ATAC-seq), histone modifications (by ChIP-seq), TF binding profiles (by ChIP-seq), and three-dimensional genomic structures (e.g., chromatin topology-associated domain boundaries and enhancer–promoter specific interactions identified through Hi-C), can directly identify active regulatory elements and their interaction networks, helping determine whether a variant could disrupt chromatin structure or gene regulation. Functional annotation features are also critical; variants in regions known to significantly affect gene expression are at a higher risk for pathogenicity. For example, variants in regions such as promoters, enhancers, 5′UTR, 3′UTR, and introns are more likely to be affected by mutations compared to other regions. Clinical and association signals from GWAS and eQTL resources, along with curated databases such as HGMD (Stenson et al., 2020) and ClinVar (Landrum et al., 2025), provide direct evidence of disease relevance for variant interpretation, as exemplified by CADD (Schubach et al., 2024), which integrates eQTL (Wong et al., 2025) and GWAS (Uffelmann et al., 2021) data to improve pathogenicity prediction. Finally, integrated functional prediction scores, which are comprehensive prediction scores based on sequence and evolutionary information, assess the functional impact of variants [e.g., CADD and EIGEN (Ionita-Laza et al., 2016)] and can serve as input features for advanced models. RegBase-PAT (Zhang et al., 2019) combines the results from 23 prediction tools [e.g., CADD, GWAVA (Ritchie et al., 2014), and DANN (Quang et al., 2015)] to train a composite model and can serve as input features for advanced models. Typically, these features (Table 2) are input into ML or DL frameworks in multimodal form, where feature interactions and pattern recognition are used to comprehensively assess the pathogenicity of noncoding variants. In the future, with further integration of multisource data, predictive models are expected to shift from “correlation” to “causality” judgments, providing more reliable variant interpretation for precision medicine.

**TABLE 2:** Predictive attributes of noncoding variant pathogenicity, associated methods, and models.

Predictive attribute	Relevance description	Indicators and sources	Representative models
Sequence context	Analyzes the physicochemical properties and compositional features of the local DNA sequence surrounding the variant	GC content, CpG islands, and nucleotide context	CADD, GWAVA, and JARVIS
Evolutionary conservation	Variants in highly conserved noncoding regions are more likely to be pathogenic	PhyloP, PhastCons, and GERP++	CADD, NCBoost, and Eigen
Epigenetic and functional genomic features	Experimental measurements of genomic functional states and 3D structural information, helping identify active regulatory elements	Chromatin accessibility, histone modifications, TF binding, and 3D genome structure	GWAVA, FINSURF, and JARVIS
Functional annotations	Determines whether a variant is located in known functional genomic regions, such as regulatory elements or noncoding RNA regions	Promoters, enhancers, 5′UTR, 3′UTR, and introns	CADD, TraP, and DYNA
Variant intolerance features	Identifies intolerant sites in core functional regions	gwRVIS, RVIS, and pLI	JARVIS and NCBoost
Population genetics	Uses population frequency data to infer natural selection pressure on variants; rare variants are more likely to be pathogenic	Allele frequency (AF), gnomAD, and 1000 Genomes	NCBoost and JARVIS
Clinical and association signals	Direct genetic evidence from disease and molecular phenotype association studies providing direct support for predictions	GWAS, eQTL, HGMD, and ClinVar	DYNA and DVAR
Integrated functional prediction scores	Comprehensive scores calculated based on multiple features; can themselves serve as powerful input for advanced models	CADD and EIGEN	regBase-PAT and CADD v1.7

This table summarizes eight key attributes commonly used to predict the pathogenicity of noncoding variants, alongside the core computational methods and representative models that leverage each attribute. Each attribute is annotated with the primary methodological approaches—traditional ML, DL, and gLMs—and representative models that integrate these features are highlighted. Detailed descriptions of the representative models are provided in Table 3. This overview serves as a comprehensive reference for understanding how diverse genomic and epigenomic features inform computational predictions of noncoding variant pathogenicity.

### Latent features as determinants of noncoding function

In contrast to explicit attributes, which rely on predefined annotations such as evolutionary conservation, epigenomic marks, chromatin states, and known regulatory elements, latent features capture higher-order sequence patterns that are not readily summarized by a limited set of handcrafted variables but are, nonetheless, critical for regulatory function. The latent features involved reflect not only the strength and direction of each transcription factor binding site but also their combinatorial syntax among multiple motifs and the sequence context from flanking sequences at different genome levels. These features influence how an allele will impact the regulatory landscape of a specific variant when considered in combination with all the background sequences surrounding it. Sequence-based learning models are intended to extract latent features from raw DNA sequence data. Under supervised training signals, they learn hierarchical representations that typically progress from local motif patterns to intermediate-range combinatorial syntax and ultimately to long-range dependencies. As a result, sequence learning models can complement traditional annotation-based integration methods when explicit annotations are deficient in content, context, or sufficiently resolution on the allele-specific basis. gLMs further extend this paradigm. Models such as DNABERT-2 (Zhou et al., 2023), Nucleotide Transformer (NT) (Dalla-Torre et al., 2025), and long-context architectures including HyenaDNA (Nguyen et al., 2023) learn generalizable sequence representations through self-supervised pretraining on large-scale genomic corpora. In downstream tasks, these pretrained representations can be transferred through fine-tuning or by comparing reference and alternative allele embeddings. As a result, gLMs help bridge the gap between explicit annotation-based modeling and sequence-derived latent representation learning, particularly in settings where detailed allele-specific resolution is required or where functional annotations remain sparse.

## The evolution of noncoding variant pathogenicity prediction

Guided by the literature search and screening strategy (Supplementary Figure S1) described in the Supplementary Material, we systematically screened the published literature and selected representative computational models for noncoding variant pathogenicity prediction. These methods can be broadly grouped into three methodological categories: integrative annotation-based models, context-dependent sequence feature models, and foundation models for noncoding variant interpretation. As shown in Table 3, the three categories differ substantially in terms of input representation, feature-learning strategy, model architecture, and output formulation and together illustrate the field’s progression from feature engineering-driven approaches to hybrid sequence-learning frameworks and, more recently, self-supervised foundation models.

**TABLE 3:** Comparative overview of computational models for noncoding variant pathogenicity prediction.

Model	Model type	Input features	Core architecture	Predicted output	Training sets	Publication year
*Integrative annotation-based models*						
CADD	ML	Functional annotations (conservation, epigenomics, TFBS, etc.)	SVM	Pathogenicity score (continuous)	Simulated DNMs and variants arisen and fixed in human populations	2014–2024
GWAVA	ML	Regulatory annotations and sequence context	RF	Functional importance score (continuous)	HGMD regulatory variants vs. 1000 genomes common variants	2014
DANN	DL	Same features as CADD	Multilayer DNN	Pathogenicity score (continuous)	Same as CADD (observed vs. simulated)	2014
FATHMM-MKL	ML	Functional annotations, sequence conservation, and protein features	Multiple kernel learning SVM	Functional effect score (continuous) and prediction confidence score	HGMD pathogenic SNVs vs. 1000 genomes	2015
ReMM	ML	Curated Mendelian noncoding mutations, conservation, and epigenomic annotations	RF + resampling	Pathogenicity score (continuous)	Hand-curated set of regulatory Mendelian mutations and derived alleles of human evolution	2016
Eigen	ML	Functional annotations, sequence conservation, and genomic context	Unsupervised learning + spectral meta-learner	Pathogenicity score (continuous)	Variants in the 1kGP without a match in dbNSFP and within 500 bp upstream of the TSS	2016
Eigen-PC	ML	Functional annotations, sequence conservation, and genomic context	Unsupervised learning + spectral meta-learner	Pathogenicity score (continuous)	Variants in the 1kGP without a match in dbNSFP and within 500 bp upstream of the TSS	2016
TRAP	ML	Genomic sequence and regulatory annotations	RF	Pathogenicity score (continuous)	75 pathogenic synonymous +402 controls	2017
DVAR	ML	Epigenomic features, sequence context, and evolutionary constraints	Unsupervised learning + multivariate Dirichlet process Mixtures	Functional score (continuous)	2 million variants randomly sampled from the 1kGP	2018
NCBoost	ML	Sequence conservation + human-specific purifying selection signals	Gradient tree boosting (XGBoost)	Pathogenicity score (continuous)	High-confidence pathogenic noncoding SNVs (HGMD-DM and ClinVar) vs. dbSNP	2019
regBase_PAT	ML	Integrated scores from 23 prediction tools (e.g., CADD, GWAVA, DANN, and EIGEN)	Gradient tree boosting (XGBoost)	Pathogenicity score (continuous)	ClinVar pathogenic vs. benign regulatory NCVs + Genomiser variants	2019
FINSURF	ML	DNA sequence features, conservation scores, regulatory element information, and variant position	SVM	Functional impact score (continuous)	HGMD noncoding pathogenic vs. ClinVar benign	2022
*Context-dependent sequence feature models*						
JARVIS	DL	gwRVIS, raw genomic sequences, and functional annotations	CNN + DNN fusion	Pathogenicity probability (continuous)	TOPMed WGS (62,784 individuals) + functional annotations	2021
*Foundation models for noncoding variant interpretation*						
NT	gLMs	Raw genomic sequences	Transformer encoder (BERT-style)	Variant effect score/regulatory prediction	Task-specific fine-tuning datasets	2021
DNABERT-2	gLMs	Raw genomic sequences	Transformer encoder (BERT-style and extended context)	Regulatory/functional variant score	Task-specific fine-tuning datasets	2022
HyenaDNA	gLMs	Raw genomic sequences	Long-context sequence model (Hyena block/SSM)	Pathogenicity probability (continuous)	GenomicBenchmarks tasks + fine-tuning on downstream datasets	2023
DYNA	gLMs + DL	Wild-type and variant sequences	Siamese neural network	Pathogenic/benign (binary)	Disease-specific variant sets (e.g., cardiac and splicing)	2025

This table summarizes the representative models for predicting the pathogenicity of noncoding variants, including integrative annotation-based models, context-dependent sequence feature models, and foundation models for noncoding variant interpretation. The models are compared in terms of model type, input features, core architecture, predicted output, training datasets, and publication year.

### Integrative annotation-based models

**FIGURE 3:**
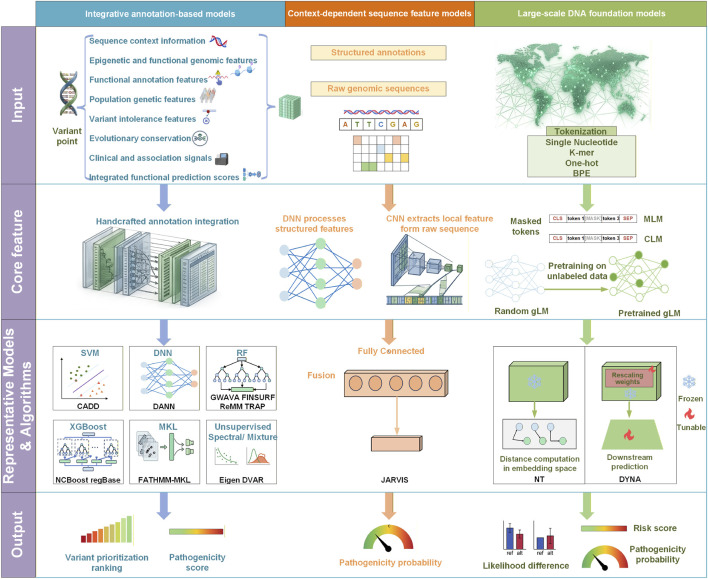
Evolution of computational paradigms for noncoding variant pathogenicity prediction. This figure presents the evolutionary framework of noncoding variant pathogenicity prediction using a horizontal stage-wise and vertical category-wise layout. Horizontally, each row represents a different stage of the analytical pipeline, including input information, feature extraction, key models and algorithms, and output format. Vertically, the figure is organized into three major methodological paradigms: integrative annotation-based models, context-dependent sequence feature models, and large-scale DNA foundation models. These three paradigms differ substantially across all stages of the workflow. Among them, integrative annotation-based models mainly rely on structured features such as evolutionary conservation, epigenomic annotations, functional annotations, population genetic information, and clinical association signals. These features are then processed using algorithms such as SVMs, RF, XGBoost, MKL, or unsupervised spectral methods to generate variant prioritization rankings or pathogenicity scores. Context-dependent sequence feature models jointly use raw genomic sequences and structured annotations as input and employ CNNs and DNNs to extract local sequence features and fuse them with annotation-derived features, ultimately outputting pathogenicity probabilities. In contrast, foundation models are trained on large-scale unlabeled genomic sequences and use different tokenization strategies together with self-supervised pretraining objectives, such as MLM and CLM, to learn transferable sequence representations. These representations can then be used for embedding-difference computation or downstream task-specific fine-tuning, producing likelihood differences, risk scores, or pathogenicity probabilities. Overall, the figure summarizes the technological progression of noncoding variant pathogenicity prediction from handcrafted feature engineering to hybrid sequence-annotation modeling and, more recently, to self-supervised pretraining with task-specific adaptation.

Integrative annotation-based models represent the earliest mainstream approach for noncoding variant pathogenicity prediction (Figure 3). In this framework, each variant is encoded as a tabular annotation feature vector. These features include sequence conservation and selective constraint, functional genomic annotations, epigenetic marks, TF binding sites, DNA methylation, population genetics information, and genomic context. Classical ML algorithms, such as support vector machine (SVM) (Zou et al., 2023) and random forest (RF), are commonly applied to process these annotation features and produce a ranked prioritization of variant pathogenicity.

The CADD (Kircher et al., 2014) model is a typical example of such annotation-integrated scoring method. The CADD model integrates over 60 functional annotations by encoding each variant as a feature vector. The derived alleles found in humans (observed in populations) and from simulated variants are used to create labels to train a linear SVM that can be used for classification prediction (Wang Y. et al., 2024). As a result, the CADD model produces a continuous deleteriousness score for genome-wide candidate variants. Across versions, CADD has undergone iterative updates: v1.4 (Rentzsch et al., 2019) and v1.6 (Rentzsch et al., 2021), which refined the annotation sources and implementation details, and v1.6 improved splicing-related signals. The latest v1.7 (Schubach et al., 2024) offers further enhancements to the representation of features, as well as additional annotations to enhance the ability to prioritize coding and noncoding regulatory variants; the CADD v1.7 also notes that scoring thresholds can be context-dependent based on the clinical perspective and the available validation resources. Deep neural networks (DNNs) have also been employed, as in DANN (Quang et al., 2015), which replaces the SVM of CADD with a DNN using the same set of features as CADD. As a result of capturing nonlinear interactions between the features, DANN provides greater model capacity for large datasets (Guo Y. et al., 2025).

Several models, including GWAVA (Ritchie et al., 2014), FINSURF (Moyon et al., 2022), ReMM (Smedley et al., 2016), and TRAP (Gelfman et al., 2017), utilize RF algorithms, which consist of many different decision trees to classify data and to perform regression. The randomness in the model from using ensembles of different decision trees as well as the fact that RFs do not require scaled features means that they work well with high-dimensional and complex datasets. Additionally, random forests provide internal measures of the contributions of each feature to the model, allowing users to interpret the model results and also perform feature selection. Thus, these models evaluate the pathogenicity of noncoding variants by using multiple annotations and, through a transformation of the multidimensional annotations (such as TF-binding sites, chromatin accessibility, histone modifications, genomic context, conservation sequencing, and local sequencing context), into a tabular feature vector. GWAVA combines various genomic annotations to output continuous scores for evaluating variant potential functionality and pathogenicity, enabling prioritization of candidate noncoding variants. FINSURF integrates 41 annotation features, producing a score that represents the probability of a variant being regulatory or pathogenic, while also providing feature-specific contributions for interpretability and identifying key disease-relevant regulatory variants. ReMM integrates evolutionary conservation, TF binding site annotations, and other features to generate ReMM scores for predicting Mendelian disease risk. TraP incorporates transcript information and genomic annotations, particularly for synonymous and intronic variants, outputting TraP scores to assess effects on splicing sites and transcript structures.

Both NCBoost (Caron et al., 2019) and regBase (Zhang et al., 2019) utilize the XGBoost algorithm, a powerful gradient-boosted tree (GBT) technique that can manage numerous complex features to identify relationships between the different features being considered when constructing a model and predicting an event. XGBoost constructs a series of decision trees and iteratively optimizes each tree’s predictive capacity, allowing the model to capture nonlinear relationships among variant features. NCBoost leverages multiple signals of natural selection to evaluate the pathogenicity of noncoding variants, integrating evolutionary conservation features, human-specific selection signals, and regional genomic characteristics to train the XGBoost model. The model outputs a pathogenicity score for each variant, which can be used to prioritize noncoding variants, highlighting those with potential deleterious effects and providing robust support for genomic data analysis. The regBase approach combines multiple models from 23 different prediction tools into a single ensemble analysis to create the regBase PAT predictive framework, which greatly improves the analytical power of this tool for utilization in clinical databases.

FATHMM-MKL (Shihab et al., 2015) leverages multiple datasets provided by ENCODE, including TFBS, chromatin accessibility (DNase-seq and FAIRE-seq), histone modifications (e.g., H3K27ac and H3K4me3), genomic segmentation states, and sequence conservation across species. FATHMM-MKL integrates these heterogeneous feature sources using a multiple kernel learning (MKL) algorithm to create a composite kernel matrix that captures the interactions between the annotations and to calculate the pathogenicity score (a measure of a variant’s likelihood of being pathogenic) for each of the variants to help rank and prioritize variants that are putatively pathogenic.

Unsupervised learning methods also play a significant role. Unlike supervised approaches, unsupervised learning does not rely on labeled data, instead uncovering intrinsic patterns in the data, which is advantageous for complex and sparsely labeled genomic datasets. For instance, Eigen (Ionita-Laza et al., 2016) uses an unsupervised spectral approach to integrate multiple functional annotations, including epigenetic marks (chromatin states, TF binding sites, and histone modifications) and evolutionary conservation (GERP and PhyloP). Eigen computes a functional score via eigenvalue decomposition of the annotation covariance matrix without requiring labels. Eigen-PC computes scores directly based on the eigenvalue decomposition of the covariance matrix. Similarly, DVAR (Yang et al., 2019) integrates diverse biological and evolutionary evidence, including ChIP-seq and DNase-seq data, conservation scores (GERP++ and PhyloP), and epigenetic data, using a Dirichlet process mixture (DPM) model to identify functional patterns and assign DVAR scores for evaluating variant functionality and pathogenicity.

### Context-dependent sequence feature models

Context-dependent sequence feature models represent a transitional approach that integrates traditional annotation features with raw genomic sequences in end-to-end sequence learning frameworks, combining structured annotations with latent sequence-derived features.

A representative method is JARVIS (Vitsios et al., 2021), which integrates gwRVIS, raw genomic sequence context, and additional structured functional annotations such as chromatin accessibility, TF binding sites, and CTCF binding sites. The CNNs process the raw sequence, extracting local sequence features through a sliding-window approach (e.g., GC content and local sequence variability) to capture latent sequence features and improve allele-specific resolution. Structured features are processed in parallel using DNNs, and the outputs from the CNN and DNN modules are fused to produce a final pathogenicity probability score, representing the likelihood of each variant being deleterious. During training, JARVIS does not rely on evolutionary conservation information, instead focusing on human lineage-specific genomic constraints and leveraging a multimodule neural network architecture to substantially improve noncoding variant pathogenicity prediction.

### Foundation models for noncoding variant interpretation

With the rapid development of large-scale self-supervised pretraining, gLMs (Shu et al., 2026) combined with clinical fine-tuning have emerged as an important technical route for predicting the pathogenicity of noncoding variants. gLMs are built on modern architectures such as transformers, Hyena, and state space models (SSMs). Representative transformer-based models include DNABERT-2 (Zhou et al., 2023) and Nucleotide Transformer (NT) (Dalla-Torre et al., 2025), whereas Hyena- and SSM-based architectures place a greater emphasis on long-context modeling and the capture of long-range dependencies, with HyenaDNA (Nguyen et al., 2023) being a representative example. Unlike traditional approaches that rely on recurrent structures or local convolutions, these models can jointly model both local and distal dependencies across sequences spanning up to millions of base pairs. Rather than depending on large amounts of manually labeled data, they are pretrained in a self-supervised manner on massive unlabeled sequence corpora, including the human reference genome, population-scale multigenome datasets, and multispecies genomes. Through such pretraining, gLMs can learn rich context-dependent representations directly from raw DNA sequences, capturing regulatory grammar, motif organization, and their potential functional consequences, thereby reducing the reliance on manually curated annotation coverage. Common pretraining objectives include masked language modeling (MLM) and causal language modeling (CLM), often combined with different tokenization strategies such as one-hot encoding, single-nucleotide embeddings, k-mers, and byte-pair encoding (BPE).

In addition, self-supervised learning turns each tokenized nucleotide position into a training signal, enabling gLMs to integrate broader pangenomic information and transfer knowledge across tasks and species. This makes them adaptable to a wide range of downstream applications, including pathogenicity prediction, gene expression prediction, and variant effect prediction. In the context of noncoding variant pathogenicity prediction, two main strategies have emerged. The first is representation-driven variant scoring, in which the sequences corresponding to the reference and alternative alleles are encoded separately, and variant effects are inferred from embedding distances or likelihood differences. NT is a representative example of this approach as it learns variant-effect features directly from sequence information without relying on conventional handcrafted annotations, making it particularly suitable for large-scale unlabeled variant datasets. The second strategy is supervised clinical fine-tuning, where a lightweight classification head is added on top of a pretrained backbone and trained using clinical or semi-clinical labels from resources such as ClinVar or HGMD to distinguish pathogenic from benign variants and output ranked risk scores or pathogenicity probabilities. DYNA (Zhan et al., 2025) exemplifies this strategy. In disease-specific settings, such as cardiovascular disorders or RNA splicing regulation, it enables more refined variant classification and pathogenicity scoring, thereby improving predictive accuracy. This route is particularly suitable for tasks in which clinical labels are closely linked to pathogenicity, as well as for small-sample, disease-specific datasets, and is more directly aligned with the practical clinical need for fine-grained pathogenic/benign discrimination.

### Comparison of the three methodological paradigms

Although the annotation-integrative, context-dependent sequence and foundation models represent successive stages of noncoding variant pathogenicity prediction, newer models are not necessarily superior to older models. They reflect different trade-offs in information sources, contextual modeling, label dependence, interpretability, and deployment cost (Table 4).

**TABLE 4:** Comparison of methodological paradigms for noncoding variant pathogenicity prediction.

Paradigm	Input	Strengths	Best use	Limitations	Practicality
Integrative annotation-based models	Structured annotations	High interpretability; low cost	Rich annotations; limited labels; clinical prioritization	Annotation coverage bias; weak allele specificity; limited distal modeling	Easy deployment
Context-dependent sequence feature models	Raw sequence ± annotations	Better allele specificity; direct sequence learning	Local sequence effects; incomplete annotations	Limited tissue specificity; weak long-range context	Moderate deployment
Foundation models with variant scoring or clinical fine-tuning	Pretrained sequence representations	Strong transferability; unlabeled data use; long-context potential	Large-scale screening; low-label transfer	Data bias; calibration issues; unstable small-data fine-tuning; high compute cost	Harder deployment

Comparison of three major methodological paradigms for noncoding variant pathogenicity prediction, namely, integrative annotation-based models, context-dependent sequence feature models, and foundation models with variant scoring or clinical fine-tuning. The table summarizes their typical inputs, major strengths, best-use scenarios, main limitations, and practical deployment characteristics.

Annotation-integrative approaches have simple architectures, fewer parameters, and strong interpretability, allowing them to retain an important role over time. Their strength lies in directly encoding prior knowledge, providing traceable evidence and clinical interpretability, although they are limited by current knowledge boundaries (Ellingford et al., 2022). Specifically, they can integrate multiple types of structured evidence, including evolutionary conservation, epigenomic annotations, population genetic information, and clinical association signals, thereby providing mechanistically traceable support for variant prioritization (Ward and Kellis, 2012). These methods are particularly advantageous when functional annotations are complete, pathogenic labels are limited, and interpretability and low deployment cost are prioritized. They are particularly well suited to clinical candidate variant prioritization and evidence tracing. The continued evolution of representative models such as CADD to version 1.7 also illustrates the enduring practical value of this paradigm (Schubach et al., 2024). However, their performance is highly dependent on the coverage and quality of existing annotation systems (Tabarini et al., 2022). Because most annotations are constructed at the regional rather than allele-specific level, they often lack sufficient allelic resolution. In addition, incomplete annotation coverage, labels that lack contextual specificity, and the limited ability to represent interactions among different regulatory effects may all cause these models to deviate from true pathogenic mechanisms (Paul et al., 2014). In particular, when tissues or cell states are mismatched, when allele-specific effects are prominent, or when long-range regulatory relationships are critical, annotation-integrative methods often fail to adequately capture the real consequences of variants. Accordingly, these approaches are especially suitable for clinical prescreening, variant prioritization, and scenarios requiring a clearly traceable evidence chain. Failure typically occurs when key pathogenic signals, such as distal enhancers, allele-specific motifs, or emerging regulatory mechanisms, are not covered by available annotations (Drubay et al., 2018).

Compared with annotation-integrative methods, context-dependent sequence models extract valuable information directly from sequences, compensating for incomplete functional annotations. Incorporating explicit annotation features can further improve allele-specific resolution, cross-genome robustness, and interpretability (Vitsios et al., 2021). However, these models have several limitations; first, they do not adequately model disease-relevant tissues or cell states, thus limiting performance when pathogenic mechanisms rely heavily on a specific biological context. Second, many of these models still rely, to some extent, on existing annotation systems and thus have not fully escaped the constraints of external prior knowledge. More importantly, their modeling capacity is often limited by sequence window size and network architecture, which weakens their ability to capture distal regulatory effects and long-range dependencies. They are particularly suitable for tasks that require direct learning of pathogenicity-related patterns from the sequence itself, such as identifying local regulatory perturbations, assessing splicing effects, and modeling allele-specific consequences. They typically fail when pathological effects depend more on the tissue context, developmental stage, or distal regulation than on the local sequence, causing overestimation of local perturbations and underestimation of a broader biological context.

The core advantage of foundation models is their ability to leverage large-scale unlabeled genomic data through self-supervised pretraining, thereby learning transferable sequence representations while reducing the reliance on manually curated annotations (Guo F. et al., 2025). When comparing foundation models with models that utilize annotation and context-dependent methods, foundation models are more equipped to absorb wide-ranging pan-genomic data and are likely to show greater performance in cross-tasks, cross-species, and long-contexts. They are particularly attractive in settings that require large-scale screening, long-sequence contextual modeling, or transfer under limited-label conditions. However, their performance is also constrained by multiple factors. First, it depends strongly on model scale, tokenization strategy, training data diversity, computational resources, input sequence length, and label quality. For example, prior studies have shown that larger models such as NT-v2 (Dalla-Torre et al., 2025) often achieve more substantial gains under task-specific fine-tuning, while performance is also highly sensitive to the choice of the tokenization strategy, such as k-mer versus BPE (Feng et al., 2025). Second, although multispecies pretraining often improves generalization across genomic tasks, increasing input length does not lead to unlimited performance gains; under constrained computational budgets, the performance may even decrease once the sequence length exceeds a certain threshold (Li et al., 2025). Third, when fine-tuned using clinical labels from databases such as ClinVar (Landrum et al., 2025) or HGMD (Stenson et al., 2020), label scarcity remains a major limitation. At the same time, imbalances in both pretraining corpora and downstream label distributions may introduce systematic bias, such as overfitting to species, tissues, cell states, or variant types that are better represented in available data, thereby reducing generalizability in real clinical settings. Moreover, a high score from a foundation model does not necessarily correspond to a directly interpretable risk probability, and score distributions may vary substantially across tasks and datasets. Therefore, clinical deployment typically still requires probability calibration and task-specific threshold setting. Finally, the interpretability remains limited, further underscoring the need for rigorous and standardized benchmark frameworks. Their typical failure mode arises when the distribution of pretraining data does not align with the true clinical task: although fine-tuning on clinical data can partially adapt the model to a specific application, limited and imbalanced clinical labels, together with remaining discrepancies between pretraining corpora and downstream tasks in both objective and biological contexts, suggest that such alignment can only partially mitigate rather than fully eliminate the pretraining–downstream distribution shift. As a result, the model may learn strong representations while still producing scores of uncertain reliability, especially under small-sample fine-tuning and cross-task transfer.

## Benchmarking metrics for predicting pathogenicity of noncoding variants

In the field of noncoding variant pathogenicity prediction, benchmarking models still face numerous challenges. The heterogeneous nature of the datasets currently being utilized, as well as the variability among the evaluative metrics used in the published literature, limits the ability to provide accurate and fair comparison of models. An established benchmarking framework is crucial to facilitate and standardize comparisons across models. Furthermore, carefully considering dataset selection and construction will ensure reliable evaluation of performance. Key factors that should be considered when selecting datasets include where positive and negative samples were obtained, number of samples, and quality of data (Table 5). Clinical pathogenicity labels are typically derived from curated databases such as ClinVar (Landrum et al., 2025) and HGMD (Stenson et al., 2020), while somatic variant datasets may incorporate COSMIC (Sondka et al., 2024). However, the number of noncoding pathogenic variants is extremely limited, and sample scarcity may lead to overfitting and constrain model generalization. Negative or control variants are usually drawn from ClinVar, gnomAD, 1kGP (Auton et al., 2015), or dbSNP (Sherry et al., 2001) population databases. The determination of whether a variant is benign or neutral in the noncoding context is not absolute; variants that are common polymorphisms are not necessarily nonfunctional, and variants that fall into the category of low effect risk are frequently found in the general population. Consequently, the choice of negative samples and filtering criteria (e.g., applying minor allele frequency thresholds or sequence/region matching) can substantially affect performance estimates. Dataset sample sizes are typically imbalanced, with pathogenic variants being far fewer than benign variants, which may bias training and evaluation metrics toward the negative class. Priority should be given to high-quality pathogenic datasets that have been clinically or experimentally validated, supported by multiple lines of evidence such as family studies or functional assays, to enhance label reliability.

**TABLE 5:** Model performance across different benchmark datasets.

ID	Compared models	Best method	AUROC	AUPRC	Test sample size (pathogenic/benign)	Database source
1	CADD, DANN, GWAVA, Funseq2, SNP, SOM, and FATHMM-MKL	CADD and DANN	0.96	0.90	683/5110	ClinVar
2		CADD and DANN	0.96	0.90	683/100000	ClinVar and 1kGP
3		GWAVA	0.78	0.01	384/100000	COSMIC and 1kGP
4	CADD, DeepSEA, CATO, EIGEN, LINSIGHT, and GWAVA	CADD	0.54	-	55453/55453	1kGP
5	FATHMM-MKL, CADD, DANN, DVAR, and 20 other models	FATHMM-MKL	0.7954	-	515/1850	ClinVar
6	FATHMM-MKL, CADD, and GWAVA	FATHMM-MKL	0.93	-	647/647	ClinVar
7	CADD v1.7, CADD v1.6, and APARENT2	CADD v1.7	-	0.381	23/3856	ClinVar
8	Trap, GERP++, and CADD	Trap	0.830	-	452/2814	ClinVar
9	DYNA, GPN, SpliceBERT, DNABERT-2, PhastCons, and PhyloP	DYNA	0.95	0.87	1200/3800	ClinVar
10	JARVIS, DeepSEA, ncER, CADD, gwRVIS, DANN, and phyloP46way	JARVIS	0.760–0.988	-	737/737	GWAS
11	JARVIS, Orion, ncER, CADD, LINDIGHT, gwRVIS, and eigenPC		0.707–0.844	-	14891/14891	gnomAD
12	NCBoost, ReMM, CADD, and GWAVA	NCBoost	0.90	0.53	70/700	HGMD-DM, ClinVar, and dbSNP
13	RegBase-PAT, ReMM, CADD, and DeepSEA	RegBase-PAT	0.83	0.62	200+/200+	ClinVar
14	FINSURF, CADD, ReMM-Genomiser, NCBoost, and Eight mainstream methods	FINSURF	0.884	0.121	62/17122	Clinvar
15	DVAR, CADD, GWAVA, DANN, and Eigen	DVAR	0.964	0.981	2713/27130	ClinVar and 1kGP
16			0.563	0.683	1867/18670	GWAS and 1kGP
17			0.611	0.725	1184/11840	GTEx and 1kGP
18			0.693	0.804	250/2500	MPRS and 1kGP

This table summarizes the comparative performance of representative models across different benchmark datasets for noncoding variant pathogenicity prediction. For each dataset, the table lists the compared models, the best-performing method, AUROC, AUPRC, sample size, and database source. The source references and detailed information for the corresponding IDs, including the original publications and datasets, are provided in Supplementary Table S1. Twenty other models are derived from a review article (Wang et al., 2023). Eight mainstream methods are derived from a study (Moyon et al., 2022). FunSeq2 (Fu et al., 2014) utilizes a weighted scoring scheme to assess variants; SNP (Li et al., 2015) is a pathogenicity scoring tool for noncoding variants based on the concept of “germline selection.” Based on the concept of “somatic selection,” SOM (Li et al., 2015) is a pathogenicity scoring tool for noncoding variants designed for specific cancer types, with its core method being the prediction of somatic mutation density. DeepSEA (Zhou and Troyanskaya, 2015) can predict the impact of noncoding variants on chromatin features. CATO (Maurano et al., 2015) is a useful tool for evaluating the functional consequences of noncoding variants on TF binding. LINSIGHT (Huang et al., 2017) uses a statistical model based on the INSIGHT framework combined with a generalized linear model to provide probabilistic scores for the potential impact of variants, including pathogenicity. APARENT2 (Linder et al., 2022) is used to evaluate the impact of variations in DNA sequences on polyadenylation signals. GERP++ (Davydov et al., 2010), PhastCons (Siepel et al., 2005), PhyloP (Pollard et al., 2010), and phyloP46way are tools for measuring evolutionary conservation scores; GPN(Benegas et al., 2023), SpliceBERT (Chen et al., 2024), and DNABERT-2 (Zhou et al., 2023) are pretrained language models. gwRVIS (Vitsios et al., 2021) is used to assess the variant tolerance in noncoding regions. Orion (Gussow et al., 2017) is a method based on human population genetics, designed to assess variants in noncoding genomic regions that are intolerant to variation.

In noncoding variant pathogenicity prediction, models trained on the same dataset can be compared directly. Model performance is typically evaluated along four dimensions: predictive accuracy, generalization, interpretability, and computational efficiency (Li et al., 2021; Wang et al., 2023; Zhang et al., 2025). Predictive accuracy and overall performance are commonly assessed using metrics such as the area under the receiver operating characteristic curve (AUROC), the area under the precision–recall curve (AUPRC), accuracy, precision, recall, and F1 score (Carrington et al., 2022; Dou et al., 2024; Yan et al., 2024; Zhao et al., 2024; Xie et al., 2025). Among these, the AUPRC is particularly informative in highly imbalanced datasets (Table 5). However, a high AUROC does not necessarily imply high precision or good biological interpretability. Empirical evaluations indicate that existing methods still have limitations in real-world applications; despite reporting high AUROC values, some tools may fail to distinguish between different alleles at the same locus. For example, GWAVA often assigns identical scores to different alleles, and CADD scores for pathogenic alleles are usually only slightly above 0.5, limiting their biological utility. When pathogenic noncoding single-nucleotide variants are extremely rare, model ranking performance decreases significantly, and the ability to distinguish alleles in close genomic proximity is reduced, performing relatively better only in promoters or ultra-conserved regions (Liu et al., 2019). When selecting datasets for model evaluation, it is important to check whether the evaluation data overlap with the model’s training set. Overlap may lead to biased assessment and overestimated performance. To prevent data leakage, independent test sets, leave-one-out validation, or cross-validation across different populations and sequencing platforms should be applied to ensure fairness and robustness. Model generalization is usually assessed using independent validation sets to examine the performance across different populations, disease types, and sequencing platforms (Cruz et al., 2023). Random splits or cross-validation under similar distributions may overestimate generalization. Therefore, rigorous non-overlapping splits combined with cross-validation across populations and sequencing platforms, or leave-one-out strategies, are recommended to systematically evaluate the robustness and generalization. Interpretability focuses on whether the model can reveal the biological signals underlying its predictions. Traditional feature-integrated models are relatively easy to interpret as researchers can trace the contribution of each annotated feature to pathogenicity prediction. In contrast, DL-based gLMs or sequence learning models, while achieving high predictive accuracy and effectively handling large-scale unlabeled variants, produce internal representations as high-dimensional embeddings or complex network weights, which are difficult to directly associate with specific biological features, resulting in lower interpretability. Consequently, feature-integrated models offer advantages in scenarios where clear mechanistic understanding or clinical decision support is required, whereas DL models are better suited for large-scale screening and complex pattern recognition, although additional methods or *post hoc* analyses are needed for mechanistic interpretation.

Computational efficiency encompasses training and inference time, memory usage, and hardware requirements, all of which are critical for large-scale genome-wide screening. Benchmarking of gLMs also includes zero-shot embeddings, where frozen pretrained models generate DNA sequence embeddings without task-specific fine-tuning (Feng et al., 2025). This approach largely avoids disease-specific training biases. Embedding pooling strategies are systematically compared to ensure fair evaluation across models. Multidataset and multitask evaluations are used to assess model generalization, and the results indicate that disease-specific fine-tuning can further improve the prediction of rare noncoding pathogenic variants.

## Translating noncoding pathogenicity predictions into clinical practice

Integrating noncoding variant pathogenicity prediction into clinical workflows requires a clinical environment aligned with the evidence chain, rather than focusing solely on algorithm deployment. WGS (Bagger et al., 2024) brings a large number of noncoding variants into clinical view; however, interpretation depends on tissue- and cell-specific contexts, and regulatory elements must be mapped to their target genes to establish disease relevance (Fulco et al., 2019). Clinical interpretation must rely on annotation and element-to-target gene evidence integration, rather than drawing conclusions based solely on a single predictive score (Ellingford et al., 2022).

In order for this environment to function properly, there are three essential components which must be present within the environment. First, a stable and standardized sequencing and variant detection pipeline is essential (Koboldt, 2020). Clinically, the same project should employ consistent workflows and parameters, ensuring that the analysis of the same sample produces reproducible and verifiable variant calls. The second component of this process is to develop an annotation and knowledge integration platform that provides an opportunity to integrate and map candidate variants (in a functionally relevant region) with an appropriate context at the tissue or cellular level, as well as the annotation frame of reference in which they would be mapped (Moore et al., 2020). Every annotation should have versioning applied for traceability within the process, which will assist in verification of annotations, as well as updating them in the future. The third principle to follow is to ensure that clinical interpretation and validation capabilities are in place (Richards et al., 2015). As predictive model scores can only be used as tools to rank candidate variants and assign weight to evidence, they cannot be relied on to determine the pathogenicity of a candidate variant. When candidate variants are sufficiently suspicious, additional functional validation—such as RNA/splicing assays (Anna and Monika, 2018), reporter gene assays (Ellingford et al., 2022), massively parallel reporter assays (MPRAs) (Tewhey et al., 2016), or CRISPR-based experiments (Fulco et al., 2019)—should be performed to reinforce evidence, ensuring that conclusions are reviewable (Tewhey et al., 2016). Furthermore, the set of interpretable noncoding regions should be predefined to avoid an unbounded interpretation space.

Predictive scores can be incorporated into the evidence chain to filter and prioritize pathogenic variants after the environment has been created. The initial filtering process is performed according to quality and variant frequency, followed by candidate prioritization among noncoding regions. The use of threshold strategies is an important step for making clinical decisions, and thresholds should not be applied in a general manner. Instead, stratified and context-specific thresholds should be defined based on the mechanistic category, available validation resources, and clinical task objectives. For example, CADD (Rentzsch et al., 2021) implements region-specific thresholds based on the score distribution of ClinVar noncoding variants across genomic regions, such as 5′UTR, introns, splicing-related elements, and 3′UTR. These thresholds serve as entry points for prioritization rather than direct pathogenicity calls. After identifying high-scoring candidates, they will undergo further evidence validation, and their outcomes will then be placed into central databases for future reference, creating a closed-loop system for demonstration of clinical utility. Data recirculation has been overlooked as an essential component of using noncoding variant prediction in clinical applications (Kapoor and Narayanan, 2023). Pathogenic labels are limited for noncoding variants; therefore, clinical practice often typically focuses on the verification of high scores through reliable annotation. These samples are not randomly selected and introduce bias. If the labels selected for review are then repeatedly used to retrain models, re-threshold, or test the model, they may continue to form a self-sustaining cycle in which actual performance may appear inflated or the model may lack optimal capability to identify variants accurately. To mitigate this risk, clinical pipelines should establish isolation mechanisms, strictly separating training and testing datasets, or use partitioning strategies to control hidden overlap, ensuring long-term model iteration and transferability (Roberts et al., 2017).

## Challenges and perspectives

### Current challenges

**FIGURE 4:**
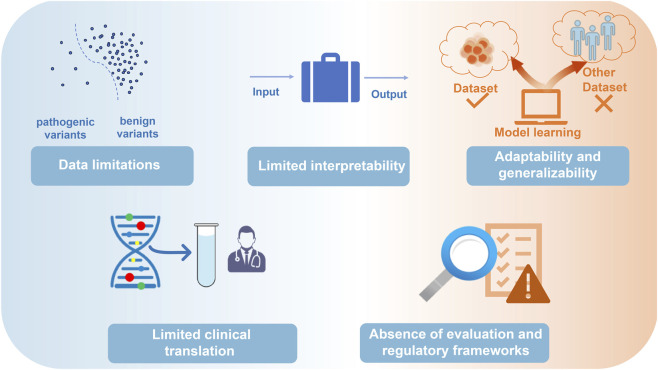
Challenges encountered in predicting the pathogenicity of noncoding variants.

First, a lack of comprehensive data is another challenge (Figure 4). Datasets differ substantially in their quality, and their internals (labels) often are insufficiently specified and suffer from a high degree of class imbalance. Additionally, there are still relatively few experimentally validated pathogenic variants as compared to benign variants, thereby limiting the effectiveness of existing supervised learning approaches. To address data scarcity, DYNA (Zhan et al., 2025) adopts a disease-specific fine-tuning strategy to improve the prediction accuracy of noncoding variants. By utilizing a Siamese neural network architecture to finetune pretrained genomic language models, DYNA produced beneficial results on small sample rare variant datasets, with a high correlation between outcomes, particularly when used in the context of noncoding pathogenic mechanisms that involve RNA splicing. In addition, the lack of high-quality, standardized “gold-standard” labels further limits the reliability of model training and evaluation. The second challenge is limited interpretability. Although DL is effective at capturing complex patterns, it often behaves like a “black box,” making it difficult to explain why a prediction is made or to connect the prediction to a specific biological mechanism. This remains a major obstacle for clinical use. Existing interpretation methods, such as attention mechanisms and feature attribution scores, still mostly operate at the level of statistical correlation and are difficult to map directly onto clear biological mechanisms or regulatory pathways. Some researchers have attempted to improve interpretability by enhancing the use of attention mechanisms or feature attribution techniques to make DL models less of a black box and also to generate model output that can be linked back to biological motifs and/or regulatory rules [e.g., ExplaiNN (Novakovsky et al., 2023)]. The third challenge of DL is limited flexibility for generalization. Many DL models are based on specific omics data, and consequently, transferring the models to other contexts can be difficult. In addition, the variability in performance of a given model across multiple datasets decreases the overall reliability and stability of that model. Transfer learning has been shown to be a potentially effective strategy to remedy these issues. For instance, Geneformer is initially trained in a self-supervised manner on large-scale single-cell transcriptomics data, enabling it to generalizable representations of gene network dynamics that can be rapidly adapted to new applications, even when new datasets are limited (Theodoris et al., 2023). The fourth challenge is the limited clinical translation: although existing models often show good predictive accuracy, there is often a lack of clear mechanistic support, making it difficult to directly incorporate these models into clinical diagnosis, genetic counseling, or treatment decision-making. In addition, integrating these models with clinical phenotypes, family history, and real-world data is still limited in scope. Finally, the lack of unified evaluative and regulatory frameworks inhibits progress within the field. Because studies are often based on disparate datasets, evaluative metrics, and validity processes, it is often impossible to fairly compare predictive models or create widely accepted benchmark systems. Therefore, these predictive models will require a greater extent of standardized evaluation and regulatory support prior to moving into large-scale clinical applications.

### Future perspectives

Looking ahead, several directions may help alleviate the challenges discussed above. First, future DL models should not focus solely on improving algorithms themselves but should also better incorporate the biological context and clinical knowledge. In other words, a closer collaboration between clinicians and algorithm researchers will be needed to define truly clinically meaningful questions, as well as appropriate model inputs and outputs. Second, multimodal learning is likely to become an important direction. Integrating genomic sequences with epigenomic information, three-dimensional genome organization, and single-cell data may provide a more comprehensive description of the biological context in which noncoding variants operate and may also help reduce the impact of data heterogeneity. Graph neural networks (GNNs) are another promising area worth further exploration. GNNs have already been applied to a range of disease-related inference and pathogenicity prediction tasks, including multitype variant classification, ncRNA/lncRNA–disease association prediction, and modeling of relationships between certain epigenetic sites and diseases (Ai et al., 2023; Farrokhi et al., 2026). However, these studies differ in both prediction targets and evaluation frameworks. For noncoding variants, one notable advantage of GNNs is their potential to extend local sequence information to higher-order regulatory networks and three-dimensional genome interactions. For example, GraphReg (Karbalayghareh et al., 2022) constructs regulatory graphs using chromatin conformation capture data and combines them with one-dimensional epigenomic signals or genomic sequences to predict gene expression, bringing enhancer–promoter interactions and distal regulatory information into a unified framework. More recently, multimodal graph models such as GNN-MAP (Yu et al., 2025) have also shown that integrating multiple layers of annotation for pathogenicity prediction is feasible, although these efforts have, so far, focused mainly on coding regions. Future graph-based models may further organize different layers of annotation into clearer and more interpretable multilayer graph structures. Combining GNNs with sequence encoders and epigenomic features may help better model distal regulatory effects and context-specific regulatory outputs. Given that graph convolutional networks (GCNs) (Hamed et al., 2025) have already been shown to effectively capture graph-structured features of DNA variant effects, their application to noncoding variant pathogenicity prediction remains highly promising. To address the shortage of labeled data, future studies may also place greater emphasis on few-shot learning and meta-learning, with the aim of extracting transferable and generalizable patterns from limited labeled samples. In addition, federated learning (FL) is also worth attention as it may enable a cross-institutional collaboration while preserving patient privacy and promoting secure genomic data sharing (Matta and Bolli, 2025). Large models have already played important roles in multiple areas of biology (Luo et al., 2025). The gLMs may also play a larger role in this field in the future. If genomic sequences are treated as a form of “biological language,” such models may provide more context-dependent information and thereby help improve our understanding of the functional effects of noncoding variants. At the same time, their potential for task-specific fine-tuning offers a new avenue for improving the accuracy of noncoding variant pathogenicity prediction. Finally, if these methods are to move closer to real-world application, model interpretability will need to be strengthened. More systematic visualization and explanation modules should be developed to identify regions of interest, connect predictions with possible biological mechanisms, and support further validation in real clinical settings. The ability to make more credible predictive models would facilitate the transition of pathogenicity predictions from research into clinical practice, which would ultimately enable earlier diagnosis, personalized treatment options, and precision medicine.

## Conclusion

Noncoding variants play important roles in human disease, yet their pathogenic interpretation remains challenging because regulatory effects are highly context-dependent and often difficult to distinguish from functional but nonpathogenic changes. This review highlights the methodological evolution of noncoding variant pathogenicity prediction, from integrative annotation-based models to context-dependent sequence feature models and, more recently to foundation-model-based approaches. Together, these paradigms have substantially improved variant prioritization by combining explicit annotations, latent sequence features, and transferable pretrained representations. Despite this progress, major obstacles remain, including limited high-quality labels, heterogeneous benchmarking datasets, insufficient interpretability, and barriers to clinical translation. Future advances will likely depend on more standardized evaluation frameworks, stronger integration of multimodal biological and clinical information, and the development of models that are not only more accurate but also more explainable and clinically actionable. With continued progress in both computational modeling and evidence-based clinical validation, noncoding variant pathogenicity prediction is expected to become an increasingly important component of precision medicine.
