# Supplementary material for: Evolving computational paradigms for noncoding variant pathogenicity prediction
Source: Front Mol Biosci. 2026 Apr 30;13:1761673. doi: 10.3389/fmolb.2026.1761673 (PMC13171353; doi:10.3389/fmolb.2026.1761673)
Supplement: Supplementary file 1 [file DataSheet1.pdf]

# Supplementary Materials

## Supplementary Table S1

**Table S1. Summary of benchmark datasets used for evaluating non-coding variant pathogenicity prediction models.** This table summarizes representative benchmark datasets used in studies of non-coding variant pathogenicity prediction and assigns each dataset a unique ID. These IDs correspond directly to the dataset IDs used in Table 4 of the main text, thereby linking the comparative results presented in the main text with the detailed dataset definitions provided in the Supporting Material. For each dataset, the table lists the variant type, pathogenic set, benign set, and corresponding references. Overall, the pathogenic sets were derived from resources such as ClinVar, COSMIC, HGMD, GWAS fine-mapped loci, GTEx, MPRS/MPRA functional datasets, evolution-based constraint sets, and manually curated Mendelian regulatory variants, whereas the benign sets were mainly obtained from ClinVar, the 1000 Genomes Project (1kGP), gnomAD, and dbSNP. Together, these datasets cover diverse categories of non-coding variation, including general non-coding variants, rare germline non-coding variants, 3'UTR variants, intronic variants, splicing variants, non-coding structural variants, high-confidence SNPs, functional regulatory variants, and fine-mapped causal regulatory variants. This table therefore provides the dataset definitions and source information underlying the benchmarking results summarized in Table 4 of the main text.

<table>
<tr><th>ID</th><th>Type</th><th>Pathogenic Set</th><th>Benign Set</th><th>References</th></tr>
<tr><td>1</td><td>Non-coding variants</td><td>ClinVar (CLNSIG code 5)</td><td>ClinVar (CLNSIG code 2)</td><td rowspan="3">(Forbes et al., 2008; Landrum et al., 2014; Auton et al., 2015; Drubay et al., 2018)</td></tr>
<tr><td>2</td><td>Non-coding variants</td><td>ClinVar (CLNSIG code 5)</td><td>1kGP, phase 3</td></tr>
<tr><td>3</td><td>Non-coding variants</td><td>COSMIC (observed ≥4 times)</td><td>1kGP, phase 3</td></tr>
<tr><td>4</td><td>Non-coding variants</td><td>Evolutionarily forbidden alleles absent in 57 non-human placental mammals that collectively span ~2.9 billion years of evolution.</td><td>1kGP, common population polymorphisms (MAF 5–15%)</td><td>(Auton et al., 2015; Rosenbloom et al., 2015; Liu et al., 2019)</td></tr>
</table>

| | | | | |
|---|---|---|---|---|
| 5 | Rare non-coding germline variants | ClinVar (pathogenic and likely pathogenic) | ClinVar | (Landrum et al., 2018; Wang et al., 2023) |
| 6 | Non-coding variants | ClinVar (pathogenic) | ClinVar (benign) | (Landrum et al., 2014; Shihab et al., 2015) |
| 7 | 3’UTR variants | ClinVar | ClinVar | (Landrum et al., 2014; Schubach et al., 2024) |
| 8 | Intronic variants | ClinVar | ClinVar | (Landrum et al., 2014; Gelfman et al., 2017) |
| 9 | Splicing variants | ClinVar | ClinVar | (Landrum et al., 2014; Zhan et al., 2025) |
| 10 | Non-coding variants | GWAS/ Mendelian/ncRNA/regulatory variants | GWAS/Mendelian/ncRNA, regulatory variants | (Wells et al., 2019; Collins et al., 2020; Vitsios et al., 2021) |
| 11 | Non-coding SVs | gnomAD v2.1-SV, dosage-sensitive, Promoter SV, UTR SV, Enhancer SV | gnomAD v2.1, Promoter SV/UTR SV/ Enhancer SV | |
| 12 | Non-coding SNVs | HGMD-DM, ClinVar | dbSNP | (Stenson et al., 2014; Landrum et al., 2016; Caron et al., 2019) |
| 13 | Non-coding SNVs | Non-coding regulatory variants causing Mendelian diseases | ClinVar | (Landrum et al., 2018; Zhang et al., 2019) |

| | | | | |
|---|---|---|---|---|
| 14 | Non-coding variants | ReMM-Genomiser model training set,+Hand-curated set of regulatory mendelian mutations and derived alleles of human evolution | ClinVar | (Smedley et al., 2016; Moyon et al., 2022) |
| 15 | Non-coding variants | ClinVar | 1kGP (MAF >0.05) | (Lonsdale et al., 2013; Auton et al., 2015; Landrum et al., 2016; Tewhey et al., 2016; Yang et al., 2019) |
| 16 | Fine-mapped causal non-coding variants | GWAS Loci, PICS (probability >10%) | 1kGP (MAF >0.05) | |
| 17 | High-confidence SNP | GTEx | 1kGP (MAF >0.05) | |
| 18 | Functional variants | MPRS | 1kGP (MAF >0.05) | |

## Literature Search and Screening Strategy

### Literature Search

We conducted a systematic literature search to identify original publications describing tools for non-coding variant pathogenicity prediction. The search was performed in Google Scholar with a cutoff date of February 2026, including only English-language publications. Search terms included: "non-coding variants", "pathogenicity prediction", "deleteriousness prediction", as well as the names of specific predictive models. The search strategy was designed to cover both broad and narrow terms to ensure comprehensive retrieval of relevant methods and tools.

### Screening Strategy

1. Inclusion Criteria

Articles were included if they met all of the following criteria:

- Original research describing computational methods or machine learning models for non-coding variant pathogenicity prediction.
- Reporting model evaluation metrics (e.g., AUROC, AUPRC) or performing validation on independent datasets.
- Providing sufficient methodological details to assess model input types and feature extraction strategies.

2. Exclusion Criteria

Articles were excluded if they met any of the following:

- Reviews, commentaries, editorials, or publications without primary computational results.
- Models that focused exclusively on coding variants.
- Studies that only predicted functional effects without evaluating pathogenicity.
- Methods that were immature, non-representative, lacked evaluation metrics, or were not reproducible.

The scope of this review was limited in terms of publication date and coverage. The purpose was not to be exhaustive, but to highlight recent and widely used state-of-the-art tools and resources.

A PRISMA flowchart (Supplementary Figure S1) illustrates the literature screening process of studies identified, screened, excluded (with reasons), and finally included in the review.

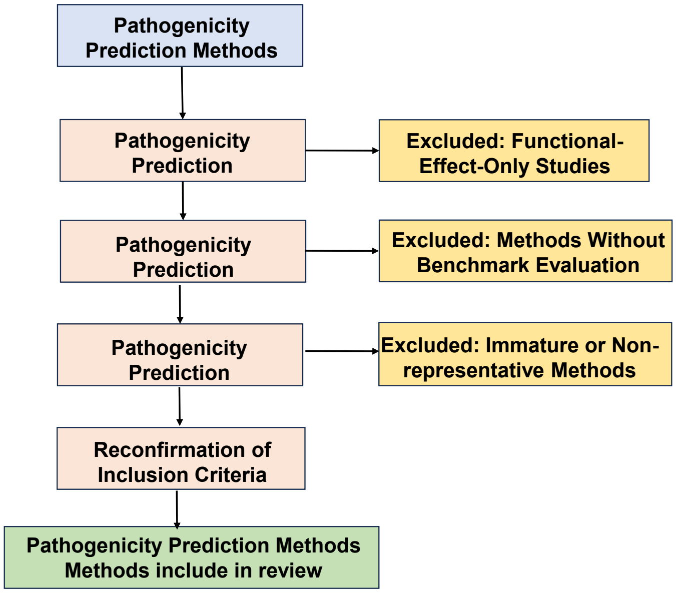


**Figure S1.** PRISMA flowchart that illustrates the stepwise process of applying inclusion and exclusion criteria to generate the final set of studies used for the literature review analysis.

The selection process, as depicted in the flowchart, includes the following steps:

**Identification (Blue):**

All relevant pathogenicity prediction methods for non-coding variants were first identified from the primary literature. This step included models reported in peer-reviewed publications and preprints with sufficient methodological details.

**Exclusion (Yellow):**

The initially identified studies were subjected to sequential exclusion criteria. Studies that only assessed functional effects without evaluating pathogenicity (Excluded: Functional-Effect-Only Studies), studies lacking benchmark evaluation (Excluded: Methods Without Benchmark Evaluation), and methods that were immature or non-representative (Excluded: Immature or Non-representative Methods) were removed during this stage.

**Inclusion (Green):**

Remaining studies were then reconfirmed against predefined inclusion criteria to ensure methodological rigor and relevance (Reconfirmation of Inclusion Criteria). The final set of studies (Pathogenicity Prediction Methods Included in Review) represents the literature selected for analysis, including representative computational models and DNA foundation models adapted for downstream pathogenicity prediction through fine-tuning.

## References

Auton, A., Abecasis, G.R., Altshuler, D.M., Durbin, R.M., Bentley, D.R., Chakravarti, A., et al. (2015). A global reference for human genetic variation. *nature* 526(7571), 68.

Caron, B., Luo, Y., and Rausell, A. (2019). NCBoost classifies pathogenic non-coding variants in Mendelian diseases through supervised learning on purifying selection signals in humans. *Genome Biol* 20(1), 32. doi: 10.1186/s13059-019-1634-2.

Collins, R.L., Brand, H., Karczewski, K.J., Zhao, X., Alföldi, J., Francioli, L.C., et al. (2020). A structural variation reference for medical and population genetics. *Nature* 581(7809), 444-451.

Drubay, D., Gautheret, D., and Michiels, S. (2018). A benchmark study of scoring methods for non-coding mutations. *Bioinformatics* 34(10), 1635-1641.

Forbes, S., Bhamra, G., Bamford, S., Dawson, E., Kok, C., Clements, J., et al. (2008). The catalogue of somatic mutations in cancer (COSMIC) Curr. Protoc. *Hum. Genet* 57(10.11), 1-10.11.

Gelfman, S., Wang, Q., McSweeney, K.M., Ren, Z., La Carpia, F., Halvorsen, M., et al. (2017). Annotating pathogenic non-coding variants in genic regions. *Nat Commun* 8(1), 236. doi: 10.1038/s41467-017-00141-2.

Landrum, M.J., Lee, J.M., Benson, M., Brown, G., Chao, C., Chitipiralla, S., et al. (2016). ClinVar: public archive of interpretations of clinically relevant variants. *Nucleic acids research* 44(D1), D862-D868.

Landrum, M.J., Lee, J.M., Benson, M., Brown, G.R., Chao, C., Chitipiralla, S., et al. (2018). ClinVar: improving access to variant interpretations and supporting evidence. *Nucleic acids research* 46(D1), D1062-D1067.

Landrum, M.J., Lee, J.M., Riley, G.R., Jang, W., Rubinstein, W.S., Church, D.M., et al. (2014). ClinVar: public archive of relationships among sequence variation and human phenotype. *Nucleic acids research* 42(D1), D980-D985.

Liu, L., Sanderford, M.D., Patel, R., Chandrashekar, P., Gibson, G., and Kumar, S. (2019). Biological relevance of computationally predicted pathogenicity of noncoding variants. *Nat Commun* 10(1), 330. doi: 10.1038/s41467-018-08270-y.

Lonsdale, J., Thomas, J., Salvatore, M., Phillips, R., Lo, E., Shad, S., et al. (2013). The genotype-tissue expression (GTEx) project. *Nature genetics* 45(6), 580-585.

Moyon, L., Berthelot, C., Louis, A., Nguyen, N.T.T., and Roest Crollius, H. (2022). Classification of non-coding variants with high pathogenic impact. *PLoS Genet* 18(4), e1010191. doi: 10.1371/journal.pgen.1010191.

Rosenbloom, K.R., Armstrong, J., Barber, G.P., Casper, J., Clawson, H., Diekhans, M., et al. (2015). The UCSC genome browser database: 2015 update. *Nucleic acids research* 43(D1), D670-D681.

Schubach, M., Maass, T., Nazaretyan, L., Roner, S., and Kircher, M. (2024). CADD v1.7: using protein language models, regulatory CNNs and other nucleotide-level scores to improve genome-wide variant predictions. *Nucleic Acids Res* 52(D1), D1143-D1154. doi: 10.1093/nar/gkad989.

Shihab, H.A., Rogers, M.F., Gough, J., Mort, M., Cooper, D.N., Day, I.N., et al. (2015). An

integrative approach to predicting the functional effects of non-coding and coding sequence variation. *Bioinformatics* 31(10), 1536-1543. doi: 10.1093/bioinformatics/btv009.

Smedley, D., Schubach, M., Jacobsen, J.O., Köhler, S., Zemojtel, T., Spielmann, M., et al. (2016). A whole-genome analysis framework for effective identification of pathogenic regulatory variants in Mendelian disease. *The American Journal of Human Genetics* 99(3), 595-606.

Stenson, P.D., Mort, M., Ball, E.V., Shaw, K., Phillips, A.D., and Cooper, D.N. (2014). The Human Gene Mutation Database: building a comprehensive mutation repository for clinical and molecular genetics, diagnostic testing and personalized genomic medicine. *Human genetics* 133(1), 1-9.

Tewhey, R., Kotliar, D., Park, D.S., Liu, B., Winnicki, S., Reilly, S.K., et al. (2016). Direct identification of hundreds of expression-modulating variants using a multiplexed reporter assay. *Cell* 165(6), 1519-1529.

Vitsios, D., Dhindsa, R.S., Middleton, L., Gussow, A.B., and Petrovski, S. (2021). Prioritizing non-coding regions based on human genomic constraint and sequence context with deep learning. *Nat Commun* 12(1), 1504. doi: 10.1038/s41467-021-21790-4.

Wang, Z., Zhao, G., Li, B., Fang, Z., Chen, Q., Wang, X., et al. (2023). Performance Comparison of Computational Methods for the Prediction of the Function and Pathogenicity of Non-coding Variants. *Genomics Proteomics Bioinformatics* 21(3), 649-661. doi: 10.1016/j.gpb.2022.02.002.

Wells, A., Heckerman, D., Torkamani, A., Yin, L., Sebat, J., Ren, B., et al. (2019). Ranking of non-coding pathogenic variants and putative essential regions of the human genome. *Nature communications* 10(1), 5241.

Yang, H., Chen, R., Wang, Q., Wei, Q., Ji, Y., Zheng, G., et al. (2019). De novo pattern discovery enables robust assessment of functional consequences of non-coding variants. *Bioinformatics* 35(9), 1453-1460. doi: 10.1093/bioinformatics/bty826.

Zhan, H., Moore, J.H., and Zhang, Z. (2025). A disease-specific language model for variant pathogenicity in cardiac and regulatory genomics. *Nature Machine Intelligence* 7(4), 661-671. doi: 10.1038/s42256-025-01016-8.

Zhang, S., He, Y., Liu, H., Zhai, H., Huang, D., Yi, X., et al. (2019). regBase: whole genome base-wise aggregation and functional prediction for human non-coding regulatory variants. *Nucleic Acids Res* 47(21), e134. doi: 10.1093/nar/gkz774.
